# Reciprocal regulation of RORγt acetylation and function by p300 and HDAC1

**DOI:** 10.1038/srep16355

**Published:** 2015-11-09

**Authors:** Qingsi Wu, Jia Nie, Yayi Gao, Peng Xu, Qijuan Sun, Jing Yang, Lei Han, Zuojia Chen, Xiuwen Wang, Ling Lv, Andy Tsun, Jijia Shen, Bin Li

**Affiliations:** 1 Department of Immunology, Anhui Medical University, Hefei, Anhui, 230032, China; 2 Department of Public Health, Anhui Medical University, Hefei, Anhui, 230032, China; 3 Key Laboratory of Molecular Virology & Immunology, Unit of Molecular Immunology, Institute Pasteur of Shanghai, Shanghai Institutes for Biological Sciences, Chinese Academy of Sciences, Shanghai, 200031, China; 4 Department of Cardiovascular Surgery, The First Affiliated Hospital of Anhui Medical University, Hefei, Anhui, 230032, China; 5 Department of Microbiology and Parasitology, Anhui Medical University, Hefei, Anhui, 230032, China; 6 Division of Rheumatology, Huashan Hospital, Fudan University, Shanghai 200040, China

## Abstract

T helper 17 (Th17) cells not only play critical roles in protecting against bacterial and fungal infections but are also involved in the pathogenesis of autoimmune diseases. The retinoic acid-related orphan receptor (RORγt) is a key transcription factor involved in Th17 cell differentiation through direct transcriptional activation of interleukin 17(A) (IL-17). How RORγt itself is regulated remains unclear. Here, we report that p300, which has histone acetyltransferase (HAT) activity, interacts with and acetylates RORγt at its K81 residue. Knockdown of p300 downregulates RORγt protein and RORγt-mediated gene expression in Th17 cells. In addition, p300 can promote RORγt-mediated transcriptional activation. Interestingly, the histone deacetylase (HDAC) HDAC1 can also interact with RORγt and reduce its acetylation level. In summary, our data reveal previously unappreciated posttranslational regulation of RORγt, uncovering the underlying mechanism by which the histone acetyltransferase p300 and the histone deacetylase HDAC1 reciprocally regulate the RORγt-mediated transcriptional activation of IL-17.

T helper 17 (Th17) cells are involved in both innate immunity and adaptive immune responses. These cells not only play critical roles in protecting against bacterial and fungal infections but are also involved in the pathogenesis of autoimmune diseases, including multiple sclerosis, arthritis, Crohn’s disease, uveitis and psoriasis^1^,^2^.

Th17 cells, which produce interleukin 17 (A) (IL-17A) and IL-17F, have been described as a separate T helper cell subset distinct from Th1, Th2 and regulatory T (Treg) cells. IL-17A and IL-17F are expressed in activated peripheral blood CD4 + T cells and induce production of proinflammatory cytokines and chemokines, including IL6 and CXCL8^3^. Transforming growth factor-β (TGF-β), IL-23 and proinflammatory cytokines (e.g., IL-1β and IL-6) are all essential for human Th17 differentiation and the expression of IL-17A, IL-17F, IL-23 receptor (IL-23R) and the retinoic acid-related orphan receptor (RORγt)^4^. The regulation of these genes is augmented by the induction of IL-21, which acts in an autocrine manner^5^. Th17 differentiation has been shown to require the transcription factors RORγt and RORα in conjunction with other essential transcription factors such as the signal transducer and activator of transcription 3 (STAT3), the aryl hydrocarbon receptor (Ahr), interferon regulatory factor 4 (IRF4), the Runt-related transcription factor 1 (Runx1), B-cell-activating transcription factor (BATF), Sox5 and c-MAF^6^,^7^,^8^,^9^. In addition, RORγt-deficient T cells inhibit Th17 cell differentiation, attenuate the expression of IL-17A and IL-17F and resist autoimmune disease. Conversely, overexpression of RORγt induces IL-17 expression and leads to more severe experimental autoimmune encephalomyelitis (EAE) than naturally occurs in wild-type mice^7^,^10^,^11^. Together, these studies suggest that RORγt is a lineage-specifying transcription factor that plays a focal deterministic role in the differentiation of Th17 cells and directs the transcriptional activation of Th17-specific genes, including IL-17A, IL-17F, IL-21 and IL-23R.

Our previous data have shown that the E3 deubiquitinase USP17 positively regulates RORγt in Th17 cells^12^. A recent study found that CNS2-deficient T cells showed decreased RORγt-driven IL-17A and IL-17F expression *in vitro*, and that CNS2-deficient mice were resistant to EAE, which may have been due to the CNS2-mediated recruitment of JmjC domain-containing protein 3 (JMJD3) and the histone acetyltransferase p300^13^. In addition, Fanpan showed that HIF1α and RORγt induced the transcription of RORγt target genes and Th17 cell differentiation through the recruitment of p300 to the IL-17A promoter^14^. However, whether p300 plays an important role in regulating RORγt and the mechanism involved remain unknown.

p300 (also known as Ep300 or KAT3B), an adenovirus E1A-associated 300- kDa protein, is a transcriptional cofactor and nuclear phosphoprotein with intrinsic acetyltransferase activity, and it regulates histones to modulate chromatin organization. In addition, p300 can regulate non-histone proteins, including nuclear transcription factors such as p53, NF-κB and Foxp3^15^,^16^,^17^. Acetylation of these transcription factors can modulate their transcriptional activity by altering their stability, subcellular localization and/or DNA-binding activity^18^.

Histone acetyltransferases and histone deacetylases reciprocally affect the steady-state levels of histone acetylation. Histone acetyltransferases typically act as transcriptional activators, and histone deacetylases, which catalyze the deacetylation of histones, generally regulate chromatin structure, modify histone and non-histone proteins, and suppress gene expression^19^,^20^. Class I histone deacetylase subfamily members include HDAC1, HDAC2, HDAC3 and HDAC8^21^; HDAC1, as a transcriptional coactivator, exerts histone deacetylation activity and plays an important role in biological processes such as cell proliferation, differentiation and cell cycle progression^20^. Recently, many studies have associated HDAC activity with diseases such as cancer, pulmonary hypertrophy and cardiac hypertrophy^22^,^23^.

Previous studies have shown that histone acetyltransferase-deacetylase complexes regulate Foxp3-mediated transcriptional suppression^24^, and p300 and the deacetylase Sirt2 have been shown to reciprocally regulate autoacetylation^25^,^26^. Furthermore, p300 and HDAC1 reciprocally regulate adenosine monophosphate-activated protein kinase (AMPK)^27^. However, whether p300 and HDAC1 reciprocally regulate RORγt acetylation and function is unclear.

Here, we report that RORγt is acetylated in Th17 cells. p300 interacts with, stabilizes and acetylates RORγt and knockdown of p300 downregulates RORγt at the protein level and decreases RORγt-mediated gene expression. p300 also promotes the RORγt-mediated transcriptional activation of IL-17. Furthermore, HDAC1 interacts with and deacetylates RORγt, leading to inhibition of RORγt-mediated IL-17 transcription. Our results reveal a previously unknown mechanism by which p300 and HDAC1 reciprocally regulate the RORγt-mediated transcriptional activation of IL-17.

## Results

### RORγt is acetylated in human Th17 cells

**Figure 1:**
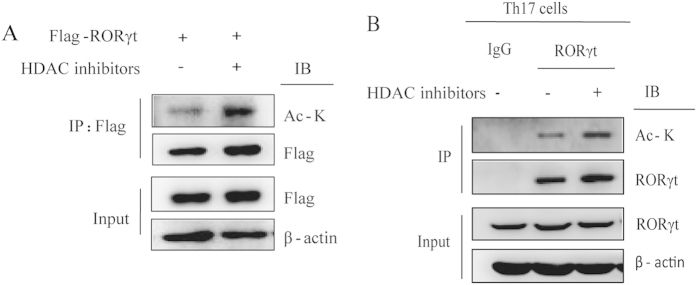
RORγt is acetylated in Th17 cells. (**A**) Flag-tagged RORγt was transfected into HEK293T cells treated either with or without HDAC inhibitors, and cell lysates were analyzed using the indicated antibodies. (**B**) Naïve CD4 + T cells were sorted from PBMCs and cultured under Th17-polarizing conditions for 7 days. The cells were treated either with or without HDAC inhibitors. Lysates from the Th17 cells were immunoprecipitated with either RORγt or control IgG antibodies and then analyzed using the indicated antibodies. Each figure is representative of >3 independent experiments.

RORγt is a master transcription factor in Th17 cells, and RORγt expression determines Th17 differentiation. First, we tested whether RORγt is acetylated in transiently transfected cells. Flag-RORγt was transfected into HEK293T cells in the presence of HDAC inhibitors, and we observed that RORγt was acetylated (Fig. 1A). Furthermore, we found that RORγt was also acetylated in Th17 cells. In addition, RORγt acetylation was significantly enhanced in the presence of HDAC inhibitors (Fig. 1B). Taken together, these data indicate that RORγt is acetylated *in vivo*.

#### p300 interacts with and stabilizes RORγt

**Figure 2:**
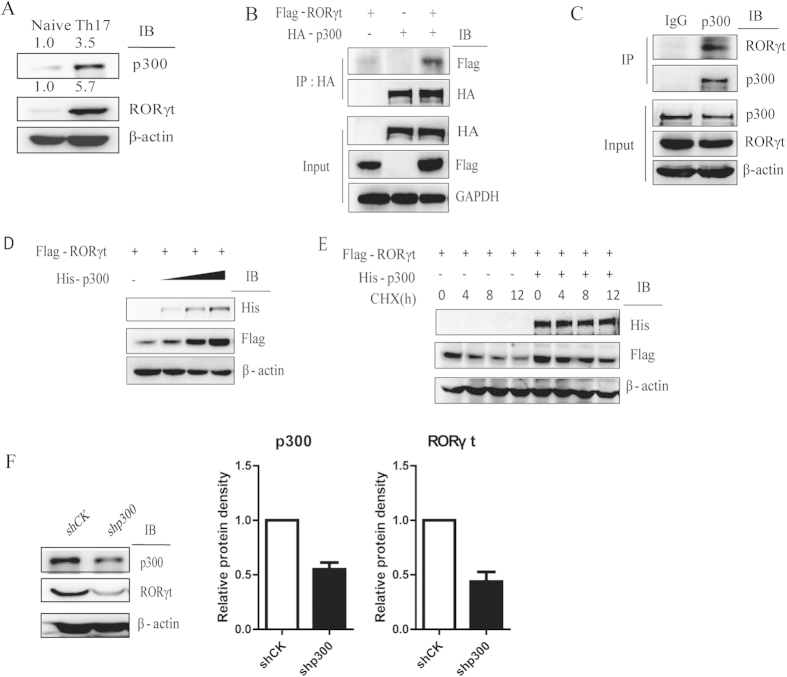
p300 interacts with and stabilizes RORγt. (**A**) Naïve CD4 + T cells were differentiated into Th17 cells *in vitro*. RORγt and p300 protein levels were analyzed by western blotting. (**B**) HEK293T cells were transiently transfected with Flag-tagged RORγt and/or HA-tagged p300. Immunoprecipitation was performed with an anti-HA antibody and analyzed by western blotting as indicated. (**C**) Endogenous interaction between p300 and RORγt in Th17 cells. (**D**) Flag-tagged RORγt was cotransfected with increasing amounts of His-tagged p300 into HEK293T cells. Cells were analyzed with western blotting. (**E**) Flag-tagged RORγt was cotransfected with His-tagged p300 into HEK293T cells. Cells were treated with CHX for the indicated periods and analyzed with western blotting. (**F**) Naïve CD4 + T cells were cultured under Th17-polarizing conditions for 7 days. Th17 cells were transduced with a lentivirus containing either shCK or shp300. Cells were treated with puromycin for 3 days and the protein levels were assessed. Each figure is representative of >3 independent experiments.

To assess whether certain acetyltransferases can upregulate RORγt-mediated transcription activation, we cotransfected 5 HATs and RORγt into HEK293T cells along with the IL-17 promoter to screen for the effects of HATs on RORγt-mediated transcriptional activation, p300 significantly upregulated RORγt-mediated transcription activation among the 5 HATs (Supplementary Figure 1). In addition, western blotting analysis comparing human naïve CD4 + T cells and Th17 cells showed that p300 protein level is higher in Th17 cells (Fig. 2A). Subsequently, to determine whether p300 associates with RORγt, coimmunoprecipitation was performed. HA-p300 and Flag-RORγt were transiently transfected into HEK293T cells, and cell lysates were analyzed using an antibody against the HA-tag. Coimmunoprecipitation between p300 and RORγt demonstrated that p300 interacts with RORγt (Fig. 2B). In addition, endogenous IP also showed that p300 interacts with RORγt in human Th17 cells (Fig. 2C). p300 localization was predominantly nuclear, and RORγt was also observed in the nucleus. These results suggest that p300 colocalizes with RORγt in HeLa cells, consistent with an interaction between p300 and RORγt (Supplementary Figure 2).

Previous studies have shown that acetylation can affect protein stabilization^17^,^28^, thus, we examined whether p300 can stabilize RORγt. We observed that a dose-dependent increase in the RORγt protein level positively correlated with the protein level of p300 (Fig. 2D). To confirm this result, we transfected Flag-RORγt into HEK293T cells either with or without His-p300 and then treated the cells with the protein synthesis inhibitor cycloheximide (CHX) at the indicated time points. Thus, we confirmed that RORγt stabilization could be positively regulated by p300 (Fig. 2E). To further test the p300-mediated stabilization of RORγt under more physiological conditions, we generated a shRNA construct targeting p300 in human Th17 cells to reduce the endogenous p300 levels and observed that knockdown of p300 decreased the level of RORγt protein (Fig. 2F).

### p300 acetylates RORγt at the K81 residue

**Figure 3:**
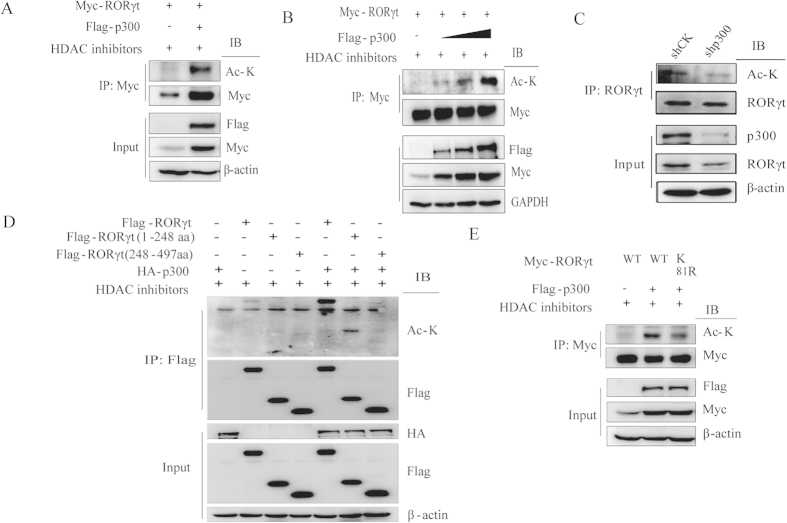
p300 acetylates RORγt at its K81 residue. (**A**) Myc-tagged RORγt and Flag-tagged p300 were cotransfected into HEK293T cells in the presence of HDAC inhibitors. Immunoprecipitation was performed using an anti-Myc antibody, and western blots were analyzed using the indicated antibodies. (**B**) Myc-tagged RORγt was cotransfected with increasing doses of Flag-tagged p300 into HEK293T cells in the presence of HDAC inhibitors. Cell lysates were immunoprecipitated with an anti-Myc antibody and analyzed by western blotting. (**C**) Naïve CD4 + T cells were differentiated into Th17 cells. Th17 cells were transduced with a lentiviral construct containing either shCK or shp300. Immunoprecipitation was performed with an anti-RORγt antibody and analyzed using the indicated antibodies. (**D**) HA-tagged p300 was cotransfected with either RORγt truncation mutants or wild-type RORγt into HEK293T cells in the presence of HDAC inhibitors. Immunoprecipitation was performed with an anti-Flag antibody and analyzed using the indicated antibodies. (**E**) Myc-tagged wild-type RORγt or the K81R mutant was cotransfected with Flag-p300 into HEK293T cells in the presence of HDAC inhibitors. Immunoprecipitation was performed with an anti-Myc antibody and analyzed using the indicated antibodies. Each figure is representative of >3 independent experiments.

p300 is an acetyltransferase with intrinsic acetyltransferase activity. Previous data have shown that p300 can acetylate transcription factors such as Foxp3 and p53^15^,^29^. To determine whether p300 can acetylate RORγt, Flag-tagged p300 and Myc-tagged RORγt plasmids were transfected into HEK293T cells then we treated the cells with HDAC inhibitors. Immunoprecipitation was performed using an anti-Myc antibody, and the results were analyzed by western blotting using the indicated antibodies. Our data indicate that p300 acetylates RORγt (Fig. 3A) and that it does so in a dose-dependent manner (Fig. 3B). In addition, to determine whether RORγt acetylation is associated with p300 in human Th17 cells, we used a shRNA construct targeting p300 in human Th17 cells to reduce the endogenous p300 level and observed that knockdown of p300 decreased the RORγt acetylation level (Fig. 3C).

Full-length RORγt contains an N-terminal domain, a hinge region domain and a ligand-binding domain. To map the region in RORγt that is acetylated by p300, either RORγt truncation mutants or wild-type RORγt was cotransfected with HA-tagged p300 into HEK293T cells. Immunoprecipitation was performed, and the results indicated that p300 acetylates RORγt on the N-terminal region (Fig. 3D). Next, we screened the N-terminal region of RORγt associated with p300 by immunoprecipitation and found that a point mutation at lysine 81 into arginine significantly decreased p300-mediated acetylation (Fig. 3E).

### HDAC inhibitors increase RORγt acetylation and RORγt-mediated IL-17 transcription

**Figure 4:**
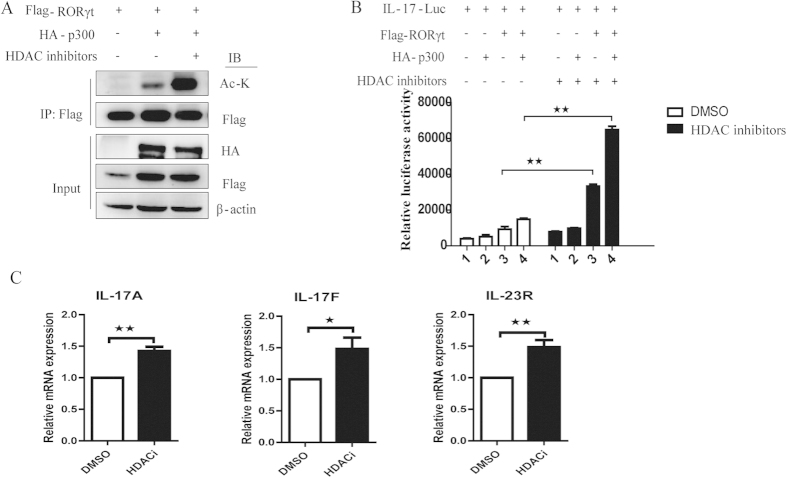
HDAC inhibitors increase RORγt acetylation and RORγt-mediated IL-17 transcription. (**A**) Flag-tagged RORγt and HA-p300 were cotransfected into HEK293T cells treated either with or without HDAC inhibitors. Cell lysates were immunoprecipitated with an anti-Flag antibody and analyzed with the indicated antibodies. (**B**) Flag**-**tagged RORγt and HA-tagged p300 were cotransfected with an IL-17 luciferase reporter into HEK293T cells in the presence of HDAC inhibitors. The cells were lysed, and luciferase activity was measured. (**C**) Th17 cells were treated with DMSO or HDAC inhibitors and analyzed using RT-PCR. Each figure is representative of >3 independent experiments. The data are presented as the mean ± SEM; *p < 0.05, **p < 0.01.

To investigate the effects of HDAC inhibitors on RORγt acetylation and RORγt-mediated transcription, we transfected Flag-tagged RORγt and HA-tagged p300 into HEK293T cells in the presence of HDAC inhibitors. We observed that HDAC inhibitors significantly increased RORγt acetylation compared to untreated control (Fig. 4A). Moreover, an IL-17A luciferase reporter was cotransfected with either HA-p300 or Flag-RORγt into HEK293T cells in either the presence or the absence of HDAC inhibitors. In this experiment, we observed that RORγt-mediated IL-17 transcription was dramatically increased in the presence of HDAC inhibitors compared to the DMSO-only control (Fig. 4B). Subsequently, when Th17 cells were treated with DMSO or HDAC inhibitors, RT-PCR analysis showed that the expression of RORγt-mediated genes were dramatically upregulated in the presence of HDAC inhibitors compared to the DMSO-only control (Fig. 4C).

### HDAC1 interacts with and deacetylates RORγt

**Figure 5:**
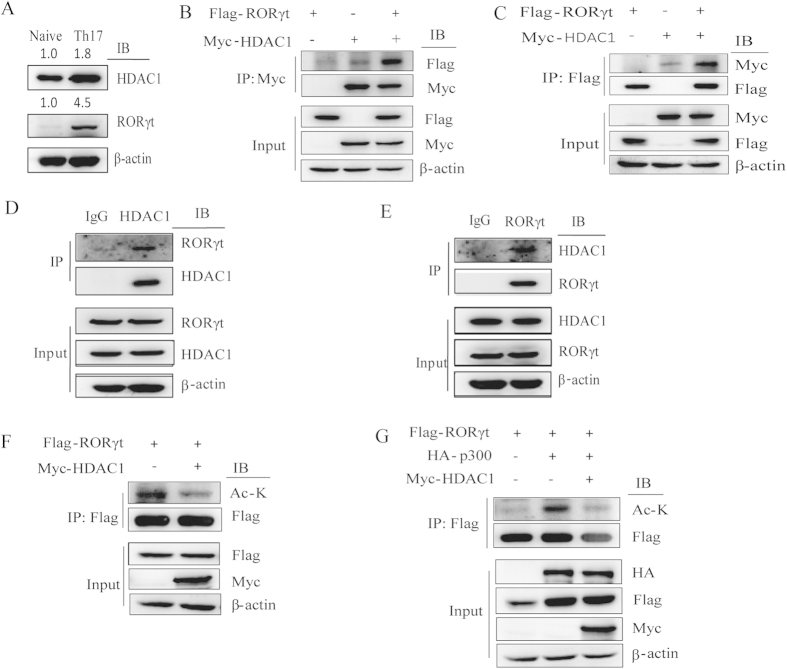
HDAC1 interacts with RORγt and deacetylates RORγt. (**A**) Naïve CD4 + T cells were differentiated into Th17 cells. HDAC1 and RORγt protein levels were analyzed by western blotting. (**B,C**) Flag-tagged RORγt and Myc-tagged HDAC1 were cotransfected into HEK293T cells. Cell lysates were coimmunoprecipitated with either anti-Myc or anti-Flag antibody and analyzed by western blotting with the indicated antibodies. (**D,E**) Endogenous interaction between HDAC1 and RORγt in human Th17 cells. (**F**) Flag-tagged RORγt and Myc-tagged HDAC1 were cotransfected into HEK293T cells. Immunoprecipitation was performed with an anti-Flag antibody and analyzed with the indicated antibodies. (**G**) Flag-tagged RORγt and HA-tagged p300 were cotransfected with Myc-tagged HDAC1 into HEK293T cells. Cell lysates were immunoprecipitated with an anti-Flag antibody and analyzed by western blotting. Each figure is representative of >3 independent experiments.

To further investigate which HDAC is responsible for the observed effects, we screened the effects of several HDACs on p300-mediated RORγt acetylation and found that HDAC1 decreased p300-mediated RORγt acetylation (Supplementary Figure 3). Subsequently, to determine the protein level of HDAC1 in naïve and Th17-polarized T cells, we used western blotting analysis and found that the protein level of HDAC1 was higher in Th17 cells compared to naïve CD4 + T cells (Fig. 5A). To verify whether HDAC1 is associated with RORγt, we transfected Myc-HDAC1 and Flag-RORγt into HEK293T cells, and the coimmunoprecipitation results showed that HDAC1 interacts with RORγt (Fig. 5B,C). In addition, endogenous IP also showed that HDAC1 interacts with RORγt in human Th17 cells (Fig. 5D,E). HDAC1 has histone deacetylase activity, and we therefore sought to determine whether HDAC1 could decrease the acetylation level of RORγt. We found that HDAC1 decreased RORγt acetylation (Fig. 5F). Furthermore, HDAC1 decreased the RORγt acetylation mediated by p300 (Fig. 5G). Collectively, these data suggest that HDAC1 interacts with and deacetylates RORγt.

### p300 and HDAC1 reciprocally regulate RORγt function

**Figure 6:**
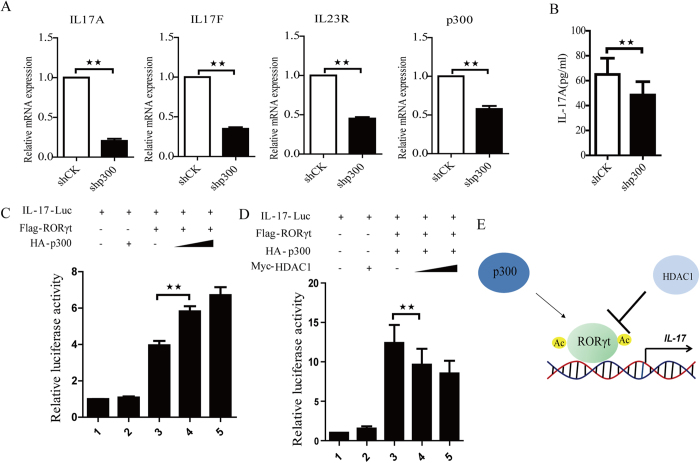
p300 upregulates RORγt-mediated IL-17 transcription, which is inhibited by HDAC1. (**A**) Naïve CD4 + T cells were sorted from PBMCs and cultured under Th17-polarizing conditions for 7 days. The Th17 cells were transduced with a lentivirus containing either shCK or shp300. The cells were then treated with puromycin for 3 days, and analysis was performed using RT-PCR. (**B**) Naïve CD4 + T cells were differentiated into Th17 cells. Th17 cells were transduced with a lentivirus containing either shCK or shp300. ELISA was performed to detect IL-17A level in Th17 cells. (**C**) Flag**-**tagged RORγt and HA-tagged p300 were cotransfected with an IL-17 luciferase reporter into HEK293T cells. The cells were lysed, and luciferase activity was measured. (**D**) Flag**-**tagged RORγt, HA-tagged p300 and Myc-tagged HDAC1 were cotransfected with an IL-17 luciferase reporter into HEK293T cells. The cells were lysed, and luciferase activity was measured. (**E**) A working model showing reciprocal regulation of RORγt acetylation and RORγt function by p300 and HDAC1. Each figure is representative of >3 independent experiments. The data are presented as the mean ± SEM; **p < 0.01.

To investigate the mechanism by which p300 regulates RORγt, we generated a shRNA construct targeting p300 to reduce the endogenous p300 levels and the RT-PCR data show that silencing p300 downregulated Th17-related genes, including IL-17A, IL-17F and IL-23R (Fig. 6A). Thus, knockdown of p300 decreases RORγt-mediated gene expression in Th17 cells. In addition, to further assess the protein levels of Th17 cytokines, we used ELISA to show that knockdown of p300 decreased the expression of IL-17A as well as RORγt (Fig. 6B). RORγt is a transcription factor that mediates IL-17 promoter activity. To determine whether p300 promotes RORγt-mediated IL-17 transcription, an IL-17 luciferase reporter was cotransfected with either HA-p300 or Flag-RORγt into HEK293T cells. Luciferase expression was analyzed and showed that p300 enhances RORγt-mediated IL-17 transcription in a dose-dependent manner (Fig. 6C). Additionally, when the IL-17 luciferase reporter was cotransfected with either HA-p300 or Flag-RORγt into HEK293T cells in the presence of Myc-HDAC1, the results indicated that HDAC1 represses p300-dependent, RORγt-mediated IL-17 transcription (Fig. 6D). Together, these data suggest that p300 enhances RORγt-mediated transcription, which is inhibited by HDAC1.

## Discussion

### The functional differentiation of CD4 +  T cells is determined by lineage-specific transcription factors

Previous studies have shown that T-bet plays a deterministic role in Th1 differentiation, whereas GATA3 and Foxp3 are important for Th2 and Treg cell differentiation, respectively^30^,^31^. Recent data have shown that RORγt is a key transcription factor that, along with other transcription factors, drives Th17 cell differentiation^9^.

Many studies have shown that post-translational modifications including acetylation, phosphorylation, methylation, sumoylation and ubiquitination affect these critical transcription factors. For example, the ubiquitin ligase Stub1 promotes Foxp3 degradation and thus negatively modulates Treg cell suppressive activity^32^, and TIP60 positively regulates ThPOK-mediated repression of eomesodermin in human CD4 + T cells^28^. Reciprocal regulation of Foxp3 acetylation and transcriptional repression occurs through the actions of the histone acetyltransferase Tip60 and the histone deacetylases HDAC7 and HDAC9^24^. The deubiquitinase USP17 positively regulates RORγt-mediated IL-17 transcription^12^. However, whether histone acetyltransferases and deacetylases regulate RORγt has remained unclear.

Here, we demonstrated that RORγt is acetylated in Th17 cells *in vivo* and that RORγt acetylation is significantly enhanced in the presence of HDAC inhibitors (Trichostatin A (TSA), nicotinamide (NAM) and EX-527). Together these HDAC inhibitors can inhibit a majority of the histone deacetylases^17^,^28^. TSA is an inhibitor for class I and II histone deacetylases, NAM is an inhibitor for class III histone deacetylases and EX-527 is a widely used inhibitor of sirtuin enzymes^33^,^34^. In a future study, we will identify which HDAC inhibitor is responsible for the observed effects.

p300 interacts with, stabilizes and acetylates RORγt at its K81 residue. Knockdown of p300 downregulates RORγt at the protein level and decreases transcription of IL-17. Previous studies have shown that many post-translational modifications have critical effects on p53 stability and function^35^. Furthermore, acetylation plays an important role in the functional regulation of p53 by p300^15^,^36^. Appropriate small-molecule inhibitors of p300 have been shown to impair Foxp3^ + ^Treg cell function and promote antitumor immunity^29^. Therefore, it will be interesting to study the acetylation and functional regulation of RORγt by p300. Previous reports have shown that p300 polyubiquitinates p53 through a ubiquitin ligase activity independent of its lysine acetyltransferase activity^37^,^38^, Stabilization of Foxp3 by p300 is associated with hyperacetylation of Foxp3, which prevents polyubiquitination and proteasomal degradation^17^. In addition, a similar mechanism for Smad7 and p53 has been previously described^39^,^40^. Therefore, whether the ubiquitin ligase activity of p300 may also regulate RORγt necessitates further investigation.

HDAC inhibitors have been shown to reduce protein levels and activity and increase the global acetylation level, resulting in altered cell proliferation, apoptosis and gene expression^41^,^42^. In this report, we provided evidence that HDAC inhibitors increase RORγt acetylation and RORγt-mediated IL-17 transcription. Recent data have shown that the histone deacetylase inhibitor ITF2357 decreases IL-6R production and RORγt expression, suppresses polarization toward Th17 cells and enhances Treg cell polarization through the IL-6-STAT3-IL17 pathway in mice^43^. The deacetylase inhibitor TSA promotes the suppressive function of Treg cells^44^. However, Zhijian showed that TSA decreases Foxp3 expression and the number of Treg cells^45^. Our results conflict with those of previous studies because of differences in factors such as treatment time, the class of the HDAC inhibitor used and the source of the specimens. Therefore, our results demonstrate that HDAC inhibitors can enhance gene transcription via inhibition of HDACs.

Protein acetyltransferases and deacetylases regulate the balance between acetylation and deacetylation of transcriptional factors, thereby affecting the expression of the involved genes. HATs, which add an acetyl group to lysine residues, are associated with gene transcription activity. However, HDACs, which attenuate acetylation levels by removing acetyl groups from their substrates, are associated with transcriptional repression^46^. In this report, we observed that HDAC1 interacts with and deacetylates RORγt and inhibits RORγt-mediated transcriptional activation, therefore, HDAC1 may be responsible for the observed effects. A previous paper showed that HDAC1, commonly considered a transcriptional corepressor, has histone deacetylase activity and represses gene transcription^20^.

RORγt is involved in autoimmune diseases. Here, we demonstrate that RORγt is acetylated, and this acetylation is reciprocally regulated by the histone acetyltransferase p300 and the histone deacetylase HDAC1. Our work suggests that p300 and HDAC1 may be novel targets for the treatment of RORγt-mediated autoimmune diseases.

## Methods

### Reagents

Anti-RORγ (sc293150), anti-p300 (sc-585), anti-Myc, anti-HA, mouse-IgG, and rat-IgG antibodies were obtained from Santa Cruz Biotechnology. Anti-actin and anti-FLAG (M2) antibodies, TSA (#052M4111V), and the protein inhibitor cocktail (#083M4021V) were obtained from Sigma-Aldrich. Anti-HDAC1 antibody (#5356S) was obtained from Cell Signaling Technology. Anti-acetyllysine antibody (ICP0380) was purchased from ImmuneChem (Canada), and EX-527 (S1541) was purchased from Selleck. Anti-CD3/CD28 Dynabeads were purchased from Invitrogen. Human IL-17A Platinum ELISA kit (BMS2017) was purchased from eBioscience. Protein A/G-agarose beads (A10001) were obtained from Abmart (China).

### Cell culture and transfection

HEK293T cells were maintained in DMEM (Hyclone) containing 10% fetal bovine serum (FBS) (131212, ExCell Biology) and transfected with polyethylenimine (PEI) reagent (23966, Polysciences) according to the manufacturer’s instructions. Cells were cultured in a 37 °C/5% CO_2_ incubator and harvested at 48 h posttransfection.

### Human cell sorting and Th17 cell differentiation assays

Peripheral blood mononuclear cells (PBMCs) were isolated from healthy donors, and CD4 + T cells were obtained using magnetic beads. Naïve T cells were sorted as CD4^ + ^CD25^low^CD45RA^high^ by FACS. Subsequently, the naïve T cells were differentiated into Th17 cells in X-VIVO15 (04-418Q, Lonza), containing 10% human blood (Gibco), 1% non-essential amino acids, 1% sodium pyruvate, 1% L-glutamine and 1% penicillin/streptomycin by stimulation with anti-CD3/CD28 dynabeads at a cell to bead ratio of 1:1 in the presence of cytokines (50 ng/ml rhIL-6 (206-IL-010, R&D), 100 ng/ml rhIL-23 (1290-IL-010, R&D), 1 ng/ml rhTGF-β (240-B-002, R&D) and 10 ng/ml rhIL-1β (201-LB-005, R&D)). Cells were cultured within 37 °C/5% CO_2_ incubator for 7 days for future use.

### Coimmunoprecipitation

At 48 h after transfection, cells were harvested, washed with ice-cold PBS, and subsequently lysed on ice for 30 minutes with protein lysis buffer (50 mM Tris-HCl (pH 7.5), 150 mM NaCl, 1 mM Na_2_EDTA, 1% NP40, 0.5% NaDOC, and 10% glycerol) containing protein inhibitors (cocktail, 1 mM Na_3_VO_4_, 10 mM NaF, and 1 mM PMSF). The cell lysates were centrifuged at 4 °C and the supernatants were immunoprecipitated by rotating for 1 h at 4 °C with antibodies and then for 1 h with proteinA/G-agarose beads. The beads were then washed with lysis buffer 4 times, and western blotting and immunoprecipitation were performed as previously described^47^.

### HAT assay

A p300-expression plasmid and a RORγt-expression plasmid were cotransfected into HEK293T cells. The cells were treated with HDAC inhibitors prior to cell harvesting (overnight treatment with 50 μM EX-527 and 4 h treatment with 1 mM NAM and 400 nM TSA). At 48 h after transfection, the cells were harvested, and immunoprecipitation was performed.

### Luciferase assays

IL-17 Luciferase reporter vectors, β-gal and plasmids were transfected into HEK293T cells. After 48 h, the cells were harvested, washed with ice-cold PBS, lysed on ice for 30 minutes with luciferase lysis buffer, and then analyzed using a dual luciferase reporter kit (Promega).

### Real-time quantitative PCR

RNA was extracted from 1 × 10^6^ cells using TRIzol (Invitrogen), and cDNA was reverse-transcribed according to the manufacturer’s instructions provided with for the SYBR reagent (PrimeScript RT reagent kit, TaKaRa). Real-time quantitative PCR was performed (SYBR Premix Ex TaqTM, TaKaRa), with β-actin expression serving as an internal control. The ABI Prism 7500 Sequence Detection System (Applied Biosystems) was used. The following primers were used for the qPCR experiments.

p300-forward: 5′- GGGAGTAAATGGAGGTGTAGG-3,

p300-reverse: 5′-AGGAAATATGGCTTGGACGAG-3′.

IL-17 A-forward: 5′-ACCAATCCCAAAAGGTCCTC-3′,

IL-17A-reverse: 5′-GGGGACAGAGTTCATGTGGT-3′.

IL-17F-forward: 5′-CCTCCCCCTGGAATTACACT-3′,

IL-17F-reverse: 5′-ACCAGCACCTTCTCCAACTG-3′.

IL-23R-forward: 5′-CATGACTTGCACCTGGAATG-3′,

IL-23R-reverse: 5′-GCTTGGACCCAAACCAAGTA-3′.

β-actin-forward: 5′-CTCTTCCAGCCTTCCTTCCT-3′,

β-actin-reverse: 5′-CAGGGCAGTGATCTCCTTCT-3′ .

### Viral transduction

PLKO.1-shCK or PLKO.1-shp300, along with VSVG and del8.9, were cotransfected into HEK293T cells using PEI. After 48 h, the viral supernatants were harvested and incubated overnight with Th17 cells in the presence of 8 μg/ml polybrene. Subsequently, the supernatants were replaced with fresh X-VIVO15 medium on day 2 and puromycin was added to the Th17 cells for 3 days to select positive clones. The following primer sequences were used.

shCK: 5′-CAACAAGATGAAGAGCACCAA-3′, shp300: 5′- CAGACAAGTCTTGGCATGGTA-3′.

### ELISA

The Human IL-17 Platinum ELISA kit was used in accordance with the manufacturer’s protocol (eBioscience).

### Confocal assay

Myc-tagged RORγt and Flag-tagged p300 were cotransfected into HeLa cells, which were fixed, permeabilized and then stained with anti-Myc or anti-p300. The cells were also stained with DAPI to visualize the nuclei. Cells were examined by confocal microscopy.

### Statistical analysis

The data are presented as the means ± SEM. Comparisons between two groups were performed using Student’s t-test. Differences were considered Statistically significant at *p < 0.05.

## Additional Information

**How to cite this article**: Wu, Q. *et al.* Reciprocal regulation of RORγt acetylation and function by p300 and HDAC1. *Sci. Rep.* **5**, 16355; doi: 10.1038/srep16355 (2015).

## Supplementary Material

Supplementary Information
