# Supplementary material for: Reciprocal regulation of RORγt acetylation and function by p300 and HDAC1
Source: Sci Rep. 2015 Nov 9;5:16355. doi: 10.1038/srep16355 (PMC4817527; doi:10.1038/srep16355)
Supplement: Supplementary Information [file srep16355-s1.doc]

Supplementary information for


Reciprocal regulation of RORγt acetylation and function by p300 and HDAC1


Qingsi Wu1,2**, Jia Nie3**, Yayi Gao3, Peng Xu4, Qijuan Sun5, Jing Yang3, Lei Han6, Zuojia Chen3, Xiuwen Wang6, Ling Lv6, Andy Tsun3, Jijia Shen1,5* and Bin Li3*

Supplementary figure 1: p300 significantly increased RORγt- mediated transcription.


IL-17-Luc

Flag-RORγt HATs
25


20


15


10


5


0


 Supplementary figure 1 | p300 significantly increased RORγt-mediated transcription.    
 Flag-tagged RORγt and HATs were cotransfected with IL-17 luciferase reporter into  
 HEK293T cells. Cells were lysed and luciferase activity were measured.


Supplementary figure 2: p300 co-localizes with RORγt in Hela cells （¨confocal ）©


DAPI	Myc-RORγt	Flag-p300	Merge


Supplementary figure 2 | p300 co-localizes with RORγt in Hela cells. Myc-tagged RORγt and Flag-tagged p300 were cotransfected into HeLa cells, which were then stained with anti-Myc or anti-p300. The cells were also stained with DAPI to visualize the nuclei.

Supplementary figure 3: HDAC1 downregulated p300-mediated RORγt acetylation


Myc-RORγt HA-p300
HDACs

IB


IP: Myc


Input
Ac-K


Myc


HA


Myc


Supplementary figure 3 |HDAC1 downregulated p300-mediated RORγt acetylation Myc-tagged RORγt and HA-tagged p300 were cotransfected into HEK293T cells in the presence of HDAC, Immunoprecipitation was performed with an anti-Myc antibody and analyzed using the indicated antibodies.
